# Modulation of mitochondrial function by the microbiome metabolite propionic acid in autism and control cell lines

**DOI:** 10.1038/tp.2016.189

**Published:** 2016-10-25

**Authors:** R E Frye, S Rose, J Chacko, R Wynne, S C Bennuri, J C Slattery, M Tippett, L Delhey, S Melnyk, S G Kahler, D F MacFabe

**Affiliations:** 1 Department of Pediatrics, University of Arkansas for Medical Sciences, Little Rock, AR, USA; 2 Arkansas Children's Research Institute, Little Rock, AR, USA; 3 Kilee Patchell-Evans Autism Research Group, Division of Developmental Disabilities, Department of Psychology/Psychiatry, University of Western Ontario, London, ON, Canada

## Abstract

Propionic acid (PPA) is a ubiquitous short-chain fatty acid, which is a major fermentation product of the enteric microbiome. PPA is a normal intermediate of metabolism and is found in foods, either naturally or as a preservative. PPA and its derivatives have been implicated in both health and disease. Whereas PPA is an energy substrate and has many proposed beneficial effects, it is also associated with human disorders involving mitochondrial dysfunction, including propionic acidemia and autism spectrum disorders (ASDs). We aimed to investigate the dichotomy between the health and disease effects of PPA by measuring mitochondrial function in ASD and age- and gender-matched control lymphoblastoid cell lines (LCLs) following incubation with PPA at several concentrations and durations both with and without an *in vitro* increase in reactive oxygen species (ROS). Mitochondrial function was optimally increased at particular exposure durations and concentrations of PPA with ASD LCLs, demonstrating a greater enhancement. In contrast, increasing ROS negated the positive PPA effect with the ASD LCLs, showing a greater detriment. These data demonstrate that enteric microbiome metabolites such as PPA can have both beneficial and toxic effects on mitochondrial function, depending on concentration, exposure duration and microenvironment redox state with these effects amplified in LCLs derived from individuals with ASD. As PPA, as well as enteric bacteria, which produce PPA, have been implicated in a wide variety of diseases, including ASD, diabetes, obesity and inflammatory diseases, insight into this metabolic modulator from the host microbiome may have wide applications for both health and disease.

## Introduction

There is growing interest in the role of the enteric microbiome in human health and disease. Alterations in the enteric microbiome and its metabolic byproducts have been implicated in variations in early brain development and behavior,^1, 2^ the development of atopic disease,^3^ psychiatric disorders including depression and anxiety,^4^ gastrointestinal disorders,^5^ diabetes^6, 7^ and obesity.^8^ In addition, the enteric microbiome modulates the immune system,^3^ metabolism^9^ and gene expression.^10, 11^

Enteric bacteria can influence host physiology through the production of short-chain fatty acids. One short-chain fatty acid, propionic acid (PPA), is ubiquitous, being derived from both endogenous and environmental sources. PPA is present or is added to foods.^12, 13, 14, 15, 16, 17, 18^ It is used in agriculture and the food industry,^19^ being a major animal silage and food preservative in wheat and dairy products.^20, 21^ PPA has been proposed to have weight loss, anti-inflammatory and cholesterol-lowering properties.^19, 22, 23^ Common enteric microbiome residents produce PPA through the fermentation of long-chain fatty acids, protein, peptides, glycoprotein and undigested carbohydrates, particularly dietary fiber and resistant starch.^24, 25^ 3-Nitropropionic acid (3NP), a chemical derivative of PPA, is a potential food contaminant and is a potent mitochondrial neurotoxin.^26^

PPA has widespread effects on cell physiology. PPA modulates fatty-acid metabolism by directly influencing adipocytes,^19, 22^ suppresses inflammation^27^ and has antibacterial effects.^28^ PPA and related short-chain fatty acids activate specific fatty-acid G-protein-coupled receptors, which have widespread effects on immunity, fatty-acid metabolism and enteric and central nervous system function,^29^ and alter gene expression.^30^

**Figure 1:**
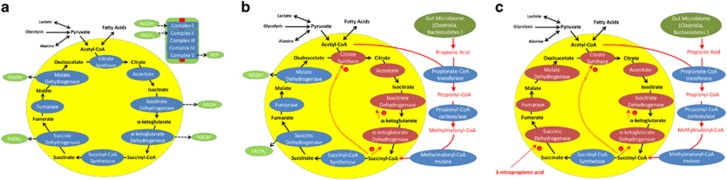
The potential effects of propionic acid on the citric acid cycle. (**a**) The citric acid cycle and associated electron transport chain complexes. The citric acid cycle is represented in the yellow circle with the enzymes of the cycle in blue ovals and the metabolites in black. The electron carriers derived from the citric acid cycle are represented in green ovals. These carriers are used by complex I and complex II of the electron transport chain (upper right corner) to produce energy. Complex V uses the energy produced by the electron transport chain to produce adenosine triphosphate (ATP), the energy carrier of the cell. (**b**) Predicted changes in citric acid cycle metabolism in the context of high propionic acid levels. Propionic acid uses a pathway that consumes acetyl-CoA to produce succinyl-CoA, an intermediate of the citric acid cycle. Thus, in the context of propionic acid, the first steps in the citric acid cycle may be bypassed and the production of nicotinamide adenine dinucleotide (NADH) may decrease. (**c**) 3-Nitropropionic acid (3NP), which could be generated from reactive nitrogen species interacting with propionic acid, inhibits succinic dehydrogenase, an important enzyme that produces a key energy carrier. This will not only decrease the production of flavin adenine dinucleotide (FADH_2_) but also the steps following, including malate dehydrogenase, another step that produces an electron carrier, NADH. This figure was adapted from Figures 1 and 2 in Frye *et al.*^36^

Propionate, the conjugate base of PPA, is an important intermediate of normal mitochondrial metabolism that is produced as the final step of odd-chain fatty-acid oxidation and isoleucine, valine and alpha-ketobutyric acid metabolism (Figure 1a). Mutations in the enzyme propionyl-CoA carboxylase block the breakdown of propionyl-CoA into methylmalonyl-CoA, causing elevations in PPA, which is associated with neurodevelopmental and gastrointestinal consequences such as propionic acidemia, a disorder that has very heterogeneous genetic variation.^31, 32, 33, 34, 35^

Autism spectrum disorder (ASD) is associated with gastrointestinal disturbances, microbiome disturbances and altered PPA metabolism.^35, 36, 37^ ASD is estimated to affect ~1.5% of children in the United States with the incidence rising.^38^ The cause(s) of ASD are still not known,^39^ and evidence for a simple genetic defect is lacking.^40^ Thus, studies suggest that the etiology of some forms of ASD involves the interaction of environmental factors, which affect broad metabolic, immune and epigenetic processes in genetically sensitive individuals.^39, 41^ Thus, the enteric microbiome is uniquely positioned as a contributory etiological environmental factor for ASD.

The significance of the enteric microbiome in ASD was demonstrated in a seminal mouse model where a probiotic significantly attenuated ASD-like behaviors.^42^ *Clostridia* spp, a broad group of normal enteric microbiome residents, which can also be highly pathogenic,^43, 44^ are associated with ASD and are a major producer of PPA.^45, 46, 47, 48, 49, 50, 51, 52^ Brief pulsed intracerebroventricular infusions of PPA into adult rodents produce ASD-like behaviors^53, 54, 55, 56, 57^ and pathophysiological abnormalities associated with ASD such as neuroinflammation,^53, 55, 56^ lipid, redox and mitochondrial abnormalities^54, 55, 58^ and epileptiform activity.^55^ Prenatal and postnatal exposure to PPA resulted in the development of ASD-related behaviors in adolescent rodents in a sexually dimorphic manner, thereby recapitulating neurodevelopmental aspects of ASD.^59, 60, 61^ Furthermore, rat pheochromocytoma cell lines exposed to PPA have altered expression of genes implicated in ASD, including those involved in synaptic function, mitochondrial function, inflammation, and learning and memory.^30^ We have proposed increased exposure or decreased breakdown of PPA to be an environmental contributor to some forms of ASD.^35, 36, 43^

Mitochondrial dysfunction through inherited and acquired impairments in carnitine metabolism may have a role in neurodevelopmental disorders including ASD.^62, 63^ A pattern of elevations in short-chain and long-chain, but not medium-chain, acyl carnitines was identified as a unique biochemical marker in the brain tissue of the PPA rodent model of ASD.^58^ A similar pattern of acyl-carnitine elevations was independently reported in 17–24% of children with ASD.^64, 65^ This subset of ASD patients is referred to as having consistent elevations in short- and long-acyl carnitines (CESLAC).^36^ CESLAC ASD children have abnormalities in mitochondrial function, particularly a deficiency in electron transport chain (ETC) complex I activity.^37, 65^ It was hypothesized that this functional deficit is caused by PPA entering the citric acid cycle (CAC) at succinyl-CoA synthetase, thereby bypassing the first four CAC enzymes, two of which create nicotinamide adenine dinucleotide, the substrate for ETC complex I (See Figure 1b).

One unresolved question is how PPA, a normal metabolic intermediate, which has the potential to be a mitochondrial fuel, and possess many proposed health benefits, can also be associated with mitochondrial dysfunction and neurodevelopmental disorders such as ASD and propionic acidemia. There are several potential possibilities. First, as hypothesized for the CESLAC subgroup, the quantity of PPA overloads the mitochondria, resulting in an imbalance in the ability of the mitochondria to efficiently produce energy (Figure 1b). Second, both ASD and propionic acidemia are associated with an unfavorable redox microenvironment. Thus, it is possible that oxidative stress causes dysfunction of mitochondrial components or the production of 3NP, a compound that is a strong inhibitor of mitochondrial function through irreversible inhibition of succinate dehydrogenase (see Figure 1c).^66^

To better understand the effect of PPA on mitochondrial function in human health and disease, we determined the ability of the mitochondria to utilize PPA and whether this ability was dependent on PPA concentration, the duration of exposure to PPA, the microenvironment redox state or the cell source. To this end, we measured mitochondrial respiratory parameters following exposure to PPA in lymphoblasoid cell lines (LCLs) derived from control children and those with autistic disorder (AD). We examined two types of AD LCLs, classified based on their mitochondrial function in previous studies, those with more normal mitochondrial function (AD-N) and those with more abnormal mitochondrial function (AD-A).^67, 68^

## Materials and methods

### Lymphoblastoid cell lines and culture conditions

LCLs derived from white males diagnosed with AD chosen from pedigrees with at least one affected male sibling (mean (s.d.) age 8.5 (3.4) years) were obtained from the Autism Genetic Resource Exchange (Los Angeles, CA, USA) or the National Institutes of Mental Health (Bethesda, MD, USA) center for collaborative genomic studies on mental disorders. In our previous studies^67, 68^ these LCLs were categorized into two different types of AD LCLs: ones with elevated mitochondrial respiratory parameters (AD-A) and the others with normal respiratory parameters (AD-N). Eight pairs of AD-N and AD-A LCLs were run with an age- and gender-matched control LCL. The sample size is based on the number of LCL pairs needed to detect differences in mitochondrial respiration in our previous laboratory experiments. Control LCLs were derived from healthy white male donors with no documented behavioral or neurological disorder or first relative with a medical disorder that could involve abnormal mitochondrial function (mean/s.d. age 8.8±3.7 years) and were obtained from Coriell Cell Repository (Camden, NJ, USA). Owing to low availability of control LCLs from children with no documented neurological disorders, we paired a single control LCL line with two AD LCL lines in one case (LCL groups are listed in Supplementary Table 1). In addition, three AD-A LCLs were paired twice with AD-N LCLs. On average, cells were studied at passage 12, with a maximum passage of 15. Genomic stability is very high at this low passage number.^69, 70^ Cells were maintained in RPMI 1640 culture medium with 15% fetal bovine serum and 1% penicillin/streptomycin (Invitrogen, Grand Island, NY, USA) in a humidified incubator at 37 °C with 5% CO_2_.

### Seahorse assay

We used the state-of-the-art Seahorse Extracellular Flux (XF) 96 Analyzer (Seahorse Bioscience, North Billerica, MA, USA) to measure oxygen consumption rate, an indicator of mitochondrial respiration, in real-time in live intact LCLs. Each run contained the three matched samples (control, AD-N and AD-A) on the same plate. The Seahorse assay (Supplementary Figure 1A), described previously,^67, 68^ provides measures of ATP-linked respiration, proton leak respiration, maximal respiratory capacity and reserve capacity.

### Redox challenge

LCLs were exposed to 0 or 10 μM of 2,3-dimethoxy-1,4-napthoquinone (DMNQ; Sigma-Aldrich, St Louis, MO, USA) for 1 h at 37 °C in a non-CO_2_ incubator before the Seahorse assay, similar to our previous studies (See Supplementary Figure 1B).^67, 68^ A 5 mg ml^−1^ DMNQ solution was diluted in DMEM XF assay media into a 10 × stock and added to cells in an XF-PS plate.

### PPA exposure

Each group of LCLs was cultured with three concentrations of PPA (0.1, 0.5 and 1 mM) for 24 or 48 h before the Seahorse assay or left untreated (0 mM; Supplementary Figure 1B). The sodium propionate is buffered with sodium bicarbonate in the culture medium to prevent changes in pH, which could cause changes in influx of PPA.^71^ As PPA is mostly disassociated at physiological pH, the effects of the PPA treatment is most likely a combination of both PPA and propionate.

### Analytic approach

A mixed-model regression^72^ was conducted via SAS version 9.3 (Cary, NC, USA) ‘glmmix' procedure. The mixed-model allowed data from the matched samples to be compared with one another. The mitochondrial respiratory parameters were the response variable with a between-group effect (for example, AD-N versus AD-A versus control) and within-group repeated factors of PPA concentration, exposure duration and reactive oxygen species (ROS) exposure as well as the interaction between these effects. For all models, random effects included the intercept. F-tests were used to evaluate significance. Planned *post hoc* orthogonal contrasts, which are t-distributed, were used to examine significant group and interaction effects. These results are reported as *P*-values in the graphs. When the effect of PPA concentration was significant, the specific PPA concentrations were compared with baseline (that is, 0 PPA) to determine whether they were significantly higher or lower than baseline. If there was an interaction with incubation time, the significant PPA concentrations were compared across incubation times and their difference was graphed to illustrate the difference between incubation times. Data were normal-distributed and variation was similar across groups. s.e. bars are provided in graphs.

### 3NP Assay

To measure the endogenous production of 3NP, a modification of the HPLC method of Muir and Majak^73^ was used. For the LCLs, the supernatant from a cell pellet extraction was used.

## Results

First, we examined the effect of PPA on control LCLs in the absence of DMNQ exposure. Next, we examined the effect of DMNQ (that is, ROS exposure) on control LCLs for the 24 and 48 h PPA incubations. Last, we compared the effect of PPA and DMNQ on the two AD groups to the control LCLs.

### Typically developing control cell lines: 24- and 48-h PPA incubation

#### ATP-linked respiration

**Figure 2:**
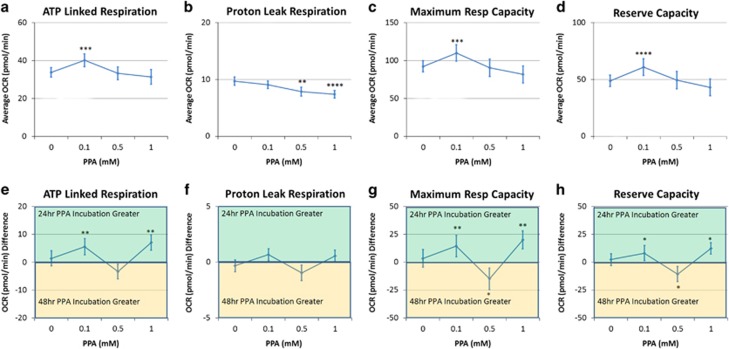
Propionic acid (PPA) increases mitochondrial function in a concentration-dependent manner. Control lymphoblastoid cell lines were incubated for either 24 or 48 in PPA and mitochondrial function was measured. Average changes in mitochondrial function across the two incubation times is shown in the top row (**a**–**d**), whereas the difference in mitochondrial function between the two incubation times is shown in the bottom row of graphs (**e**–**h**). ATP-linked respiration (**a**) as well as maximal respiratory capacity (**c**) and reserve capacity (**d**) were elevated at 0.1 mM PPA relative to no PPA exposure with this effect greater with 24 h exposure as compared with 48 h exposure (**e**, **g**, **h**). Statistical significance levels: *P⩽0.05, **P⩽0.01, ***P⩽0.001, ****P⩽0.0001.

A concentration effect (F(3,202)=10.79, *P*<0.0001) was due to an elevated ATP-linked respiration at 0.1 mM (Figure 2a). A concentration by time interaction (F(3,202)=4.53, *P*=0.001; Figure 2e) resulted from a greater ATP-linked respiration for 24 h as compared with 48 h at 0.1 and 1 mM.

#### Proton leak respiration

A concentration effect (F(3,202)=7.69, *P*<0.0001) was due to a decreased proton leak respiration at 0.5 and 1mM PPA (see Figures 2b and f).

#### Maximal respiratory capacity

A concentration effect (F(3,202)=14.56, *P*<0.0001) was due to an elevated maximal respiratory capacity at 0.1 mM (Figure 2c). A concentration by time interaction (F(3,202)=5.56, *P*=0.001) resulted from maximal respiratory capacity being greater for 0.1 and 1 mM and lower at 0.5 mM for 24 h as compared with 48 h (Figure 2g).

#### Reserve capacity

A concentration effect (F(3,202)=14.11, *P*<0.001) was due to an elevated reserve capacity at 0.1 mM (Figure 2d). A concentration by time interaction (F(3,202)=5.09, *P*<0.005) resulted from reserve capacity being higher at 0.1 and 1 mM and lower at 0.5 mM for 24 h as compared with 48 h (Figure 2h).

### Typically developing control cell lines: the effect of ROS

The effect of DMNQ on 24 and 48 h PPA incubation was analyzed separately, although the results of both the 24- and 48-h PPA incubation are both described in this section together. The effect of PPA without DMNQ described above was not repeated.

#### ATP-linked respiration

**Figure 3:**
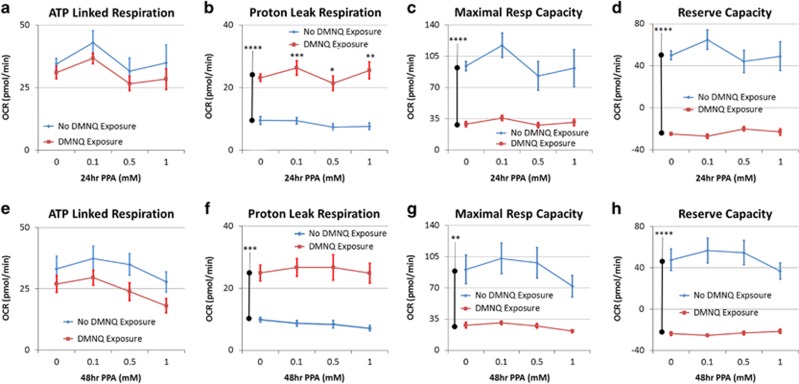
Oxidative stress reduces mitochondrial function and attenuates the effect of 24 h (**a***–***d**) and 48 h (**e**–**h**) propionic acid (PPA) incubation. Control lymphoblastoid cell lines were pretreated with 10 μM of 2,3-dimethoxy-1,4-naphthoquinone (DMNQ) for 1 h to increase intracellular reactive oxygen species before the mitochondrial assay after PPA had been washed from the cell culture. Overall DMNQ pretreatment increased proton leak respiration (**c**, **g**) and reduced maximal respiratory capacity (**d**, **h**) and reserve capacity (**e**, **g**). In addition, DMNQ pretreatment eliminated the positive effect of propionic acid on mitochondrial function seen without DMNQ pretreatment. The bars adjacent to the data lines represent the overall significant difference. Statistical significance levels: *P⩽0.05, **P⩽0.01, ***P⩽0.001, ****P⩽0.0001.

There was no effect of DMNQ (Figures 3a and e).

#### Proton leak respiration

DMNQ increased proton leak respiration for 24 h (F(1,7)=156.19, *P*<0.0001) and 48 h (F(1,7)=51.41, *P*<0.0005; Figures 3b and f). For 24 h there was a DMNQ by concentration interaction (F(3,204)=5.44, *P*=0.001) as, with DMNQ, proton leak respiration was higher at 0.1 and 1mM and lower at 0.5 mM.

#### Maximal respiratory and reserve capacity

DMNQ lowered maximal respiratory capacity at 24 h (F(1,7)=25.37, *P*=0.001) and 48 h (F(1,7)=25.51, *P*=0.002) and reserve capacity at 24 h (F(1,7)=66.52, *P*<0.0001) and 48 h (F(1,7)=64.40, *P*<0.0001; Figures 3c, d, g and h). A DMNQ by concentration interaction was found for maximal respiratory capacity at 24 h (F(3,204)=7.60, *P*<0.0001) and 48 h (F(3,204)=4.43, *P*<0.005) and reserve capacity at 24 h (F(3,204)=18.39, *P*<0.0001) and 48 h (F(3,204)=10.73, *P*<0.0001) because of the fact that PPA concentration had no effect on these respiratory parameters when exposed to DMNQ as compared with the modulatory effect PPA demonstrated without DMNQ exposure.

### Autistic cell lines

Here we compare the difference in metabolism between the AD LCLs and controls, considering the studies above on PPA metabolism in controls as a baseline. The two PPA incubation times of 24 and 48 h are reported separately.

### Autistic cell lines: the effect of 24-h PPA incubation as compared with controls

#### ATP-linked respiration

**Figure 4:**
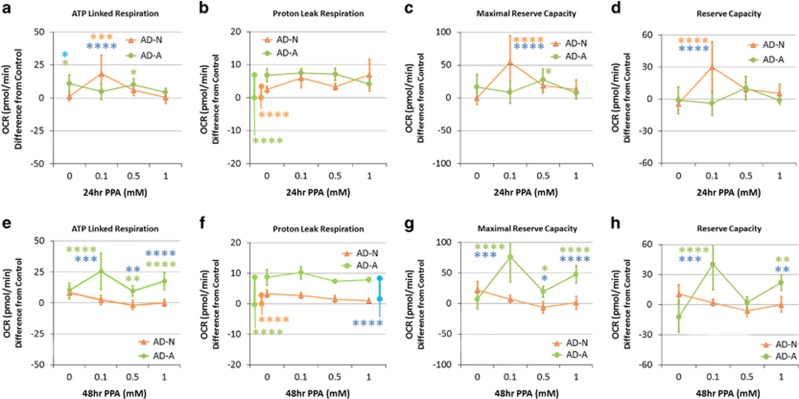
Propionic acid (PPA) enhances mitochondrial function in autistic lymphoblastoid cell lines (LCLs) over and above the effect it has on control LCLs in a concentration and exposure time-dependent manner. ATP-linked respiration, maximal respiratory capacity and reserve capacity were enhanced over and above control values at 0.1 mM for the autistic disorder with normal mitochondrial function (AD-N) LCLs with 24 h PPA incubation (**a**, **c**, **d**), whereas all of these mitochondrial parameters were enhanced at 0.1 and 1 mM for the autistic disorder with abnormal mitochondrial function (AD-A) LCLs with 48-h PPA incubation (**e**, **g**, **h**). Proton leak respiration was also significantly increased above control values for the autistic LCLs as compared with controls (**b**, **f**). The bars adjacent to the data lines represent overall significant differences. The color of the stars and bars represents the specific comparisons. Green represents the difference between AD-A and Control LCLs. Orange represents the difference between AD-N and Control LCLs. Blue represents the difference between the AD-N and AD-A LCLs. Statistical significance levels: *P⩽0.05, **P⩽0.01, ***P⩽0.001, ****P⩽0.0001.

A group by concentration interaction (F(6,325)=4.26, *P*<0.0005) was due to ATP-linked respiration being higher for (a) AD-A as compared with control and AD-N with no PPA, (b) AD-N as compared with control and AD-A at 0.1 mM, (c) AD-A as compared with the control at 0.5 mM (Figure 4a).

#### Proton leak respiration

A group effect (F(2,325)=25.11, *P*<0.0001) was driven by a higher overall proton leak respiration in AD-N and AD-A as compared with Control (Figure 4b).

#### Maximal respiratory capacity

A group by concentration interaction (F(6,325)=4.91, *P*<0.0001) was due to maximal respiratory capacity being higher for (a) AD-N as compared with Control and AD-A at 0.1 mM and (b) AD-A as compared with Control at 0.5 mM (Figure 4c).

#### Reserve capacity

A group by concentration interaction (F(6,325)=4.69, *P*=0.0001) was due to AD-N demonstrating a higher reserve capacity as compared with control and AD-A at 0.1 mM (Figure 4d).

### Autistic cell lines: the effect of 48-h PPA incubation as compared with controls

#### ATP-linked respiration

The group by concentration interaction (F(6,325)=3.68, *P*<0.005) was due to ATP-linked respiration being higher in AD-A as compared with control and AD-N for all of the PPA concentrations (Figure 4e).

#### Proton leak respiration

A group effect (F(2,325)=143.17, *P*<0.0001) was driven by higher proton leak for AD-N and AD-A as compared with the control and AD-A as compared with AD-N (Figure 4f).

#### Maximal respiratory capacity

A group by concentration interaction (F(6,325)=6.12, *P*<0.0001) was driven by a higher maximal respiratory capacity for AD-A as compared with control and AD-N for all PPA concentrations (Figure 4g).

#### Reserve capacity

A group by concentration interaction (F(6,325)=6.74, *P*<0.0001) was driven by higher reserve capacity for AD-A as compared with control and AD-N at 0.1 and 1 mM (Figure 4h).

### Autistic cell lines: the ROS effect on 24-h PPA incubation

**Figure 5:**
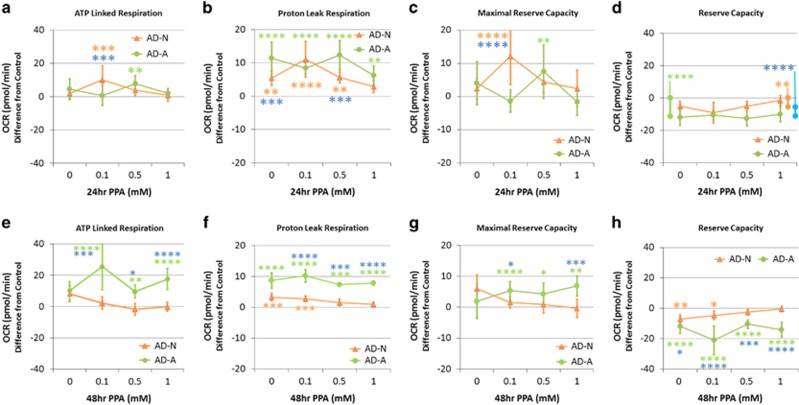
Oxidative stress reduces mitochondrial function and reverses the enhancement effect of propionic acid (PPA) on mitochondrial function in autistic lymphoblastoid cell lines (LCLs). LCLs were pretreated with 10 μM of 2,3-dimethoxy-1,4-naphthoquinone (DMNQ) for 1h to increase intracellular reactive oxygen species before the mitochondrial assay after PPA had been washed from the cell culture. Although PPA increased ATP-linked respiration (**a**, **e**) and maximal respiratory capacity (**c**, **g**) above control values in a manner similar to the increase seen without DMNQ exposure, the increase in proton leak respiration was relatively greater (**b**, **f**), resulting in a net depletion in reserve capacity as compared with control LCLs (**d**, **h**). This is particularly true for the autistic disorder with abnormal mitochondrial function (AD-A) LCLs with a 48-h PPA incubation where the PPA concentrations that previously caused enhancement now result in a depletion in reserve capacity (**h**). The color of the stars and bars represents the specific comparisons. Green represents the difference between AD-A and control LCLs. Orange represents the difference between autistic disorder with normal mitochondrial function (AD-N) and control LCLs. Blue represents the difference between the AD-N and AD-A LCLs. Statistical significance levels: *P⩽0.05, **P⩽0.01, ***P⩽0.001, ****P⩽0.0001.

*ATP-linked respiration.* A group by concentration interaction (F(6,342)=2.74, *P*<0.05) was due to ATP-linked respiration being higher for (a) AD-N as compared with control and AD-A at 0.1 mM and (b) AD-A as compared to Control at 0.5 mM (Figure 5a).

#### Proton leak respiration

A group by concentration interaction (F(6,342)=3.62, *P*<0.005) was due to proton leak respiration being higher for both (a) AD-N and AD-A as compared with control and AD-A as compared with AD-N in the absence of PPA, (b) both the AD-N and AD-A as compared with Control at 0.1 mM, (c) both AD-N and AD-A as compared with control and AD-A as compared with AD-N at 0.5 mM, and (d) AD-A as compared with control at 1 mM (Figure 5b).

#### Maximal respiratory capacity

A group by concentration interaction (F(6,342)=4.32, *P*<0.0005) was due to maximal respiratory capacity being higher for (a) AD-N as compared with control and AD-A at 0.1 mM and (b) AD-A as compared with control at 0.5 mM (Figure 5c).

#### Reserve capacity

A group effect (F(6,342)=44.94, *P*<0.0001) was due to a lower reserve capacity for both AD-N and AD-A as compared with controls and AD-A as compared with AD-N (Figure 5d).

### Autistic cell lines: the ROS effect on 48-h PPA incubation

#### ATP-linked respiration

A group by concentration interaction (F(6,344)=3.34, *P*<0.01) was driven by AD-A having higher ATP-linked respiration for all PPA concentrations as compared with control and AD-N (Figure 5e).

#### Proton leak respiration

A group by concentration interaction (F(6,344)=3.77, *P*=0.001) was due to proton leak being higher for (a) both AD-N and AD-A as compared with control with no PPA; (b) AD-A for all of the PPA concentrations as compared with control and AD-N; and (c) AD-N as compared with control at 0.1 mM (Figure 5f).

#### Maximal respiratory capacity

A group by concentration interaction (F(6,344)=2.95, *P*<0.01) was due to maximal respiratory capacity being higher for AD-A as compared with control for all of the PPA concentrations and AD-N at most PPA concentrations (Figure 5g).

#### Reserve capacity

A group by concentration interaction (F(6,344)=3.95, *P*<0.001) was due to reserve capacity being lower for (a) both AD-N and AD-A as compared with control and AD-A as compared with AD-N with no PPA; (b) AD-A for all of the PPA concentrations as compared with control and AD-N; and (c) AD-N as compared with control at 0.1 mM (Figure 5h).

### Measurement of 3-nitropropionic acid

3NP was measured intracellularly and in the media for one cell line (17255) with exposure to 0.1 mM PPA for 48 h and 10 μM DMNQ. Supplementary Figure 2C demonstrates that 3NP does not exist in the intracellular sample. Similar findings were found for the cellular media.

## Discussion

We believe this is the first systematic investigation of the effects of PPA on mitochondrial function in human cell lines. PPA is important in human health for several reasons: (a) it is a metabolic mediator of gut–host interactions; (b) it is commonly found in the diet; (c) it may have a role in common medical diseases such as obesity, diabetes and inflammatory bowel disease; (d) it is significantly elevated in inborn errors of metabolism; and (e) it may have a role in the pathophysiology of ASD and other psychiatric disorders. This study examined LCLs from children with AD as mitochondrial dysfunction in a subset of children with ASD has been linked to the overproduction of PPA, and enteric bacteria that produce PPA are over-represented in individuals with ASD and are linked to ASD behaviors.^36, 44, 65^

Despite the association of PPA with disease, PPA is paradoxically both a normal mitochondrial fuel and potential toxin. It was hypothesized that several factors such a PPA concentration, incubation time, microenvironment redox state and cell type may alter the manner in which PPA is metabolized and utilized. Thus, these parameters were systematically altered to gain insight into the effects of PPA on the mitochondria. Below we summarize our findings as well as discuss the potential importance of PPA in health and disease.

### The effect of PPA on control cell lines

PPA has a positive effect on mitochondrial function in LCLs derived from healthy children with this effect dependent on PPA concentration, incubation time and microenvironment redox state. PPA at 0.1-mM increases ATP-linked respiration, maximal respiratory capacity and reserve capacity—parameters linked to energy production and resilience to metabolic stressors. This effect was greatest when LCLs were incubated for 24 h. Longer exposure and higher PPA concentrations were not beneficial for control LCLs and, in fact, higher PPA concentrations increased proton leak consistent with an increase in ROS at the inner mitochondrial membrane.

In humans, PPA concentration is highest in the ascending colon (30 mM kg^−1^) and lowest in the ileum (1.5 mM kg^−1^). PPA is absorbed into the portal vein where its concentration is ~88 μM l^−1^.^74^ In this study, the peak utilization of PPA occurred at 100 μM/L PAA, which is very similar to portal vein concentration, suggesting that optimal mitochondrial utilization of PPA in controls occurs at physiological concentrations. The fact that higher PPA concentrations increase ROS is consistent with studies that find increased ROS in propionic acidemia patient fibroblasts.^75^

PPA is metabolized to propionyl-CoA that enters the CAC as succinyl-CoA. At physiological concentrations, this adds substrates to the CAC and enhances mitochondrial function. However, high levels of succinyl-CoA, especially for a prolonged period of time, can inhibit CAC enzymes and overutilize acetyl-CoA, both of which might result in mitochondrial dysfunction, presumably by reducing the production of nicotinamide adenine dinucleotide, resulting in a decrease in ETC complex I function (Figure 1b).^36, 65^ This provides an example of how tightly tuned metabolic systems can become imbalanced by either too little or too much of a metabolite.

Increasing intracellular ROS had an overall detrimental effect on the mitochondria and disrupted the utilization of PPA as a fuel source. First, increased ROS resulted in a detrimental shift in several important mitochondrial parameters, including a reduction in both maximal respiratory capacity and reserve capacity and an increase in proton leak respiration. The beneficial effects of PPA on mitochondrial function were not observed in the context of an oxidized intracellular microenvironment.

Increased ROS can have detrimental effects on mitochondrial function in several ways. An oxidized intracellular microenvironment can cause dysfunction of mitochondrial enzymes, including aconitase^64^ and ETC complex I and III, and can damage lipids, including cardiolipin,^54, 58^ and proteins, producing reactive nitrogen species (RNS)^55^ and disrupting protein folding.^76^ RNS, when present, can react with PPA to produce 3NP, a compound that strongly inhibits mitochondrial function through irreversible inhibition of succinate dehydrogenase (Figure 1c).^66^ Inhibition of succinate dehydrogenase shuts down the CAC from the succinate onward. Succinate dehydrogenase is one of four CAC reactions that produce an electron carrier that fuels the ETC (Figure 1c). In addition, the malate production, which is necessary to produce an electron carrier to fuel the ETC, will also be inhibited. Thus, in the context of increased ROS, PPA could have a significant detrimental effect on the CAC beyond the effect of ROS itself. The reduction in the important parameters of ATP production would support this notion. However, our measures did not support 3NP being produced in any significant quantity, as ATP-linked respiration was not significantly diminished and 3NP was not found intracellularly or in the media. This suggests that the elevation in proton leak caused enough ETC dysfunction to drive down maximal respiratory capacity.

### The effect of PPA on cell lines derived from autistic children

The effect of PPA on mitochondrial function in the two subsets of AD LCLs was similar in several ways. Like controls, AD LCLs demonstrated a marked increase in ATP-linked respiration, maximal respiratory capacity and reserve capacity at 0.1 mM PPA, but this response was significantly more robust than the control LCLs. For AD-N LCLs, this effect was significant at 24 h, whereas this effect was most robust for the AD-A LCLs at 48 h. AD-A LCLs also demonstrated a significant increase in these parameters at 1mM PPA at 48 h.

These data suggest that, in a favorable redox state, LCLs derived from individuals with AD can utilize PPA as an energy source over and above the ability of healthy LCLs in a concentration- and time-dependent manner. This suggests that compensatory adaptations have occurred in mitochondrial or other metabolic pathways to allow the AD cells to utilize PPA as a fuel. Previously, we have suggested that certain individuals with ASD may have adapted to utilize PPA in the CAC, with mitochondrial dysfunction potentially occurring during periods of high PPA exposure (Figure 1b).^36, 65^ This may be due to increased exposure to PPA in the children from whom these LCLs were derived. Indeed, *Clostridia* spp is an enteric producer of PPA, which is over-represented in the microbiome of some children with ASD,^45, 46, 47, 48, 49, 50, 51, 52^ and PPA concentrations are elevated in the stool from individuals with ASD.^77^ Interestingly, removing wheat and/or diary from the diet has been associated with improved behaviors in some children with ASD.^78^

In our previous studies, we demonstrated that AD-A LCLs differ from the AD-N LCLs in regards to their unusually high ATP production and a vulnerability to ROS such that reserve capacity is depleted at lower concentrations of DMNQ as compared with AD-N and control LCLs.^67, 68^ As these LCLs already have a higher ATP production, it may take higher concentrations of PPA over longer periods of time to further increase ATP production-related processes. Alternatively, the patients from whom these LCLs were derived may have a particular over-representation of *Clostridia* spp with overproduction of PPA, and these cells may have already adapted to utilize high levels of PPA. Future studies should be on mitochondrial function in tissues (immune, gut enterocytes and so on) in patients in which their microbiome is also examined.

The increased ATP production seen in the AD-A LCLs at 48 h was most robust at 0.1mM PPA and 1mM PPA, with a slightly less response at 0.5 mM PPA. As the LCLs were of the same passage and are plated at the same time, kept in the same incubator for the same length of time and as the PPA was diluted from the same stock and LCLs of different concentrations were run on the same Seahorse plate during the same assay, the experimental conditions were essentially identical except for the PPA concentration. This suggests that the difference in moderate ATP production was probably due to the dose-dependent effects of PPA.^19, 22, 30, 35, 43^ Further investigation will likely be helpful to better understand the dose-dependent effect of PPA on metabolic pathways.

### The effect of PPA on LCLs derived from autistic children in an oxidative microenvironment

Increased ROS altered the ability of the AD LCLs to utilize PPA to enhance mitochondrial function. With 24 h PPA incubation, ATP-linked respiration and maximal respiratory capacity were higher at 0.1 mM PPA for AD-N LCLs and 0.5 mM PPA for AD-A LCLs, as compared with control LCLs, similar to a non-oxidized microenvironment. However, proton leak respiration was also higher for AD-A and AD-N LCLs at 0.1 and 0.5 mM PPA as compared with controls, resulting in a significant reduction in reserve capacity in both AD-A and AD-N LCLs as compared with controls for all PPA concentrations. Thus, even though PPA increased the production of ATP, in the oxidative microenvironment the increase in proton leak respiration negated this effect, resulting in an overall depression in reserve capacity.

With 48 h incubation, the AD-A LCLs demonstrated higher ATP-linked respiration and maximum respiratory capacity as compared with both the AD-N and control LCLs, but proton leak respiration was also elevated. Like the 24 h incubation, the increase in proton leak respiration negated any effect of increased energy production resulting in a reduction in reserve capacity across all PPA concentrations for AD-A LCLs.

Thus, overall, an oxidized microenvironment detrimentally influenced mitochondrial metabolism of the AD LCLs, leading to a depression in reserve capacity. The reduction in reserve capacity may be particularly significant as a reduction in reserve capacity has been linked to aging,^79^ heart disease^80^ and neurodegenerative disorders.^81, 82^ Reserve capacity is important for protecting the cell from acute ROS increases, but once reserve capacity is exhausted, cell vulnerability is increased and viability is reduced.^83^ As reserve capacity is an index of cell and mitochondrial health as well as resilience, these data suggest that an oxidized intracellular microenvironment disproportionally affects the mitochondria of AD LCLs as compared with control LCLs and that the addition of PPA further exacerbates this effect.

Children with ASD have unfavorable redox metabolism in plasma,^84^ brain^64^ and immune cells.^68^ Similar processes have been observed in the PPA rodent model.^55^ In addition, AD LCLs may be particularly vulnerable in creating RNS. Indeed, 3-nitrotyrosine, a marker of protein damage and RNS, has been reported to be elevated in the ASD brain,^64, 85, 86^ LCLs^68^ and plasma.^84^ In addition, dysregulation of nitric oxide production results in increased production of peroxynitrite, a very unstable RNS,^87, 88, 89^ in children with ASD. As described above, RNS can react with PPA to create 3NP, which has direct adverse effects on the function of the CAC (Figure 3c). Thus, AD cells may be particularly vulnerable to this mechanism of mitochondrial dysfunction. However, the data suggest that the majority of the effect is because of increased proton leak rather than a reduction in ATP production, suggesting that inhibition of the CAC in the context of increased ROS is probably a minor factor contributing to the reduction in reserve capacity. Our undetectable measurement of 3NP in a subset of LCLs subjected to high levels of oxidative stress confirms this notion.

### Connection with the enteric microbiome

Enteric microbiome residents, particularly, *Clostridia* spp and Bacteroidetes genera, are producers of PPA,^43, 44^ particularly when provided wheat-based substrates.^25, 90^ PPA has been shown to modulate cell signaling,^91^ cell interactions,^92^ gene expression,^93^ immune function^94^ and neurotransmitter synthesis and release,^95^ and to influence mitochondrial^31^ and lipid^58, 96^ metabolism in both human studies and translational animal models.^19, 22, 35^ Interestingly, neurodevelopmental abnormalities, including ASD features, are seen in individuals with impaired PPA metabolism.^31, 32, 33^ Furthermore, PPA has been shown to modulate ASD gene expression through epigenetic mechanisms, including genes involved in neurotransmitter systems, neuroplasticity, neurodevelopment, neuronal cell adhesion molecules, inflammation, oxidative stress, lipid metabolism and mitochondrial function.^30, 35, 44^ This study demonstrates further evidence of a link between the enteric microbiome and the human host through mitochondrial function and demonstrates a mechanism in which the enteric microbiome can modulate host physiology.

### Connection with clinical patients

Several studies point to over-representation of *Clostridia* spp in children with ASD,^45, 46, 47, 48, 49^ particularly those with regression^50, 51^ and/or those with gastrointestinal symptoms at or before ASD symptom onset.^52^ Treatment with vancomycin, an antibiotic aimed at decreasing *Clostridia* spp, transiently decreases ASD symptoms.^97^ This suggests that *Clostridia* spp could have a connection with the etiology of ASD in some cases potentially through mitochondrial dysfunction. Furthermore, it may offer some explanation of observations of improvement in ASD symptoms following dietary reduction in refined carbohydrates,^78^ which may reduce substrate for enteric bacteria to produce PPA, and improvement of ASD symptoms with carnitine, which may improve mitochondrial function and the degradation of PPA.^63^

CESLAC patients demonstrated unfavorable markers of redox metabolism.^65^ Given that data from this study suggest that ROS compromises mitochondrial health in AD LCLs, especially when exposed to PPA, this would suggest that increased levels of ROS found in ASD patients could result in PPA derived from microbiome bacteria having a detrimental rather than favorable effect on the mitochondria. The influence of ROS on the ability of the mitochondria to utilize PPA also suggests a mechanism by which *Clostridia* spp could be related to a healthy microbiome in some cases and detrimental effect in other cases. Whereas typically developing individuals do not normally have increases in ROS, individuals with disease, such as inflammation from an inflection or inflammatory bowel disease, could manifest increased ROS in the gut. Thus, in this manner, PPA-producing enteric organisms could provide health benefits in healthy individuals while resulting in mitochondrial dysfunction in those with disease. Furthermore, certain bactericidal antibiotics (for example, β-lactams, quinolones and aminoglycosides) and proton pump inhibitors (omeprazole), which are routinely given to pediatric and obstetric patients, could further cause ROS via mitochondrial dysfunction.^98, 99^

## Conclusion

PPA is a ubiquitous compound derived from endogenous metabolism and the environment, including the enteric microbiome and common dietary sources. The effect of PPA appears to be both tissue- and dose-specific, and has underappreciated implications in both healthy and diseased states, including ASD. In this first systematic investigation of the effects of PPA on mitochondrial function in human cell lines, we demonstrated that the effect of PPA on the mitochondria is concentration-, exposure duration- and microenvironment ROS-dependent. The effects of PPA are extremely broad, influencing many biochemical processes (receptors, mitochondria, lipids, gene expression). Whether these findings translate in other cell types (that is, neurons, glia, liver and gut), involve the whole organism (that is, animal models or human patients), or host microbiota, have developmental windows of sensitivity, are permanent or reversible and/or are direct or compensatory is currently unknown. This study does suggest the need for further evaluation of potential benefits and risks regarding the widespread use of PPA in food, medicine and agriculture,^23, 100^ and its possible interaction with many neuropsychiatric conditions with mitochondrial dysfunction.^35, 36, 101^ Nonetheless, this study provides a new understanding of how the enteric microbiome may modulate host physiology at a variety of levels and may contribute to the etiology of ASD.
