# Supplementary figures and images for: Modulation of mitochondrial function by the microbiome metabolite propionic acid in autism and control cell lines
Source: Transl Psychiatry. 2016 Oct 25;6(10):e927–. doi: 10.1038/tp.2016.189 (PMC5290345; doi:10.1038/tp.2016.189)

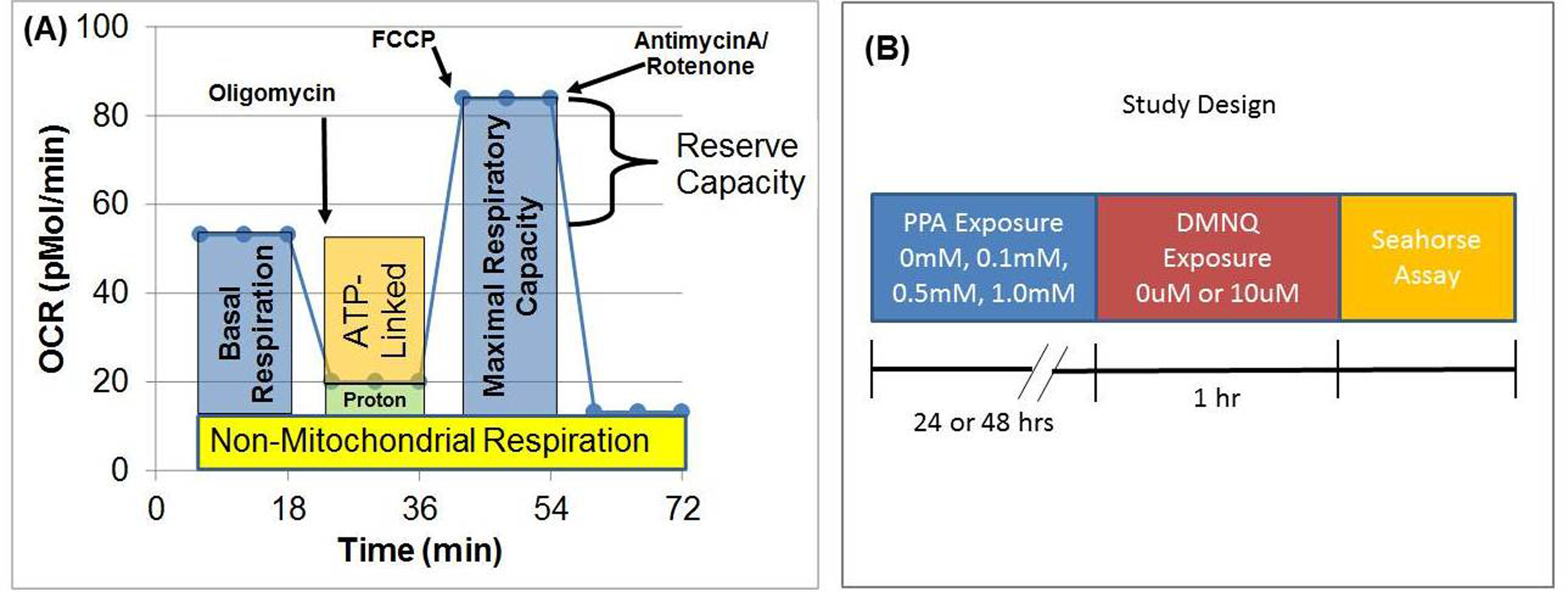

Supplement: Supplementary Figure S1 [file tp2016189x1.tif]
