# Supplementary material for: Modulation of mitochondrial function by the microbiome metabolite propionic acid in autism and control cell lines
Source: Transl Psychiatry. 2016 Oct 25;6(10):e927–. doi: 10.1038/tp.2016.189 (PMC5290345; doi:10.1038/tp.2016.189)
Supplement: Supplementary Figure Legends [file tp2016189x3.docx]

**Supplementary** **Figure Captions**

Supplementary Figure 1. Seahorse Assay and Experimental Timeline. (A) Oxygen consumption rate (OCR) is measured to determine mitochondrial activity. Three OCRs are measured over an 18 minute period to determine mitochondrial activity. Inhibitors are added to determine several parameters of mitochondrial activity. Basal Respiration is initially determined as the difference between baseline OCR and non-mitochondrial OCR. Oligomycin, which is a complex V inhibitor, is added to determine the portion of Basal Respiration which is **ATP-Linked Respiration** and **Proton Leak Respiration**. Carbonyl cyanide-p-trifluoromethoxyphenyl-hydrazon (FCCP), a protonophore, is added to collapse the inner membrane gradient, driving the mitochondria to respire at its maximal rate. This can be used to determine **Maximal Respiratory Capacity**. Antimycin A and rotenone, which are inhibitors of complex III and I, are added to stop mitochondrial respiration in order to determine the non-mitochondrial respiration. **Reserve Capacity** is calculated as the difference between Basal Respiration and Maximal Respiratory Capacity. (B) Timeline for the experiment. Lymphoblastoid cell lines (LCLs) are exposed to one of three concentrations of propionic acid or not exposed as a baseline control. Following propionic acid exposure, the LCLs are exposed to DMNQ for 1hr in order to increase reactive oxygen (ROS) species or not exposed to increased ROS. The Seahorse assay is performed after these exposures.

Supplementary Figure 2. Measurement of 3-Nitropropionic (3NP) acid by High Performance Liquid Chromatography. (A) and (B) demonstrate the chromatogram for a 3NP standard of 10ng and 100ng, respectively; peak seen at 4.29mins and 4.18mins respectively. (C) Intracellular chromatogram demonstrates a peak at 5.32mins without a peak near the standards. (D) Intracellular sample spiked with standard demonstrates the 3NP standard at 4.82mins, demonstrating that the 3NP peak is separate from the peak in the sample.

| **Supplementary Table 1.** Lymphobastoid Cell Lines used in this study. Note three types of cell lines were used with two types of Autistic Disorder cell lines which were characterized in our previous studies. | | | | | | | | |
| --- | --- | --- | --- | --- | --- | --- | --- | --- |
| **Controls** | | | **AD-N Subgroup** | | | **AD-A Subgroup** | | |
| **Cell ID** | **Source** | **Age (y)** | **Cell ID** | **Source** | **Age (y)** | **Cell ID** | **Source** | **Age (y)** |
| GM09659 | Coriell | 4 | 04C24363 | NIMH | 4 | 1393306 | AGRE | 3 |
| GM17255 | Coriell | 6 | 02C10054 | NIMH | 6 | 01C08594 | NIMH | 7 |
| GM16007 | Coriell | 12 | 05C38988 | NIMH | 12 | 1165302 | AGRE | 13 |
| GM18054 | Coriell | 5 | 03C15992 | NIMH | 5 | 01C08495 | NIMH | 4 |
| GM11626 | Coriell | 13 | 008404 | AGRE | 13 | 1165302 | AGRE | 13 |
| GM09642 | Coriell | 7 | 01C08367 | NIMH | 7 | 01C08594 | NIMH | 7 |
| GM09642 | Coriell | 7 | 04C27439 | NIMH | 7 | 02C09713 | NIMH | 7 |
| GM09380 | Coriell | 6 | 01C08022 | NIMH | 5 | 01C08495 | NIMH | 4 |

Coriell = Coriell Cell Repository (Camden, NJ, USA); NIMH = National Institutes of Mental Health Biorepository (Bethesda, MD, USA); AGRE= Autism Genetic Resource Exchange Biorepository (Los Angeles, CA, USA)
